# Supplementary material for: Towards a kingdom of reproductive life - the core sperm proteome
Source: Reproduction. 2025 May 10;169(6):e250105. doi: 10.1530/REP-25-0105 (PMC12070448; doi:10.1530/REP-25-0105)
Supplement: Supplementary file 8 [file supplementary_materials.pdf]

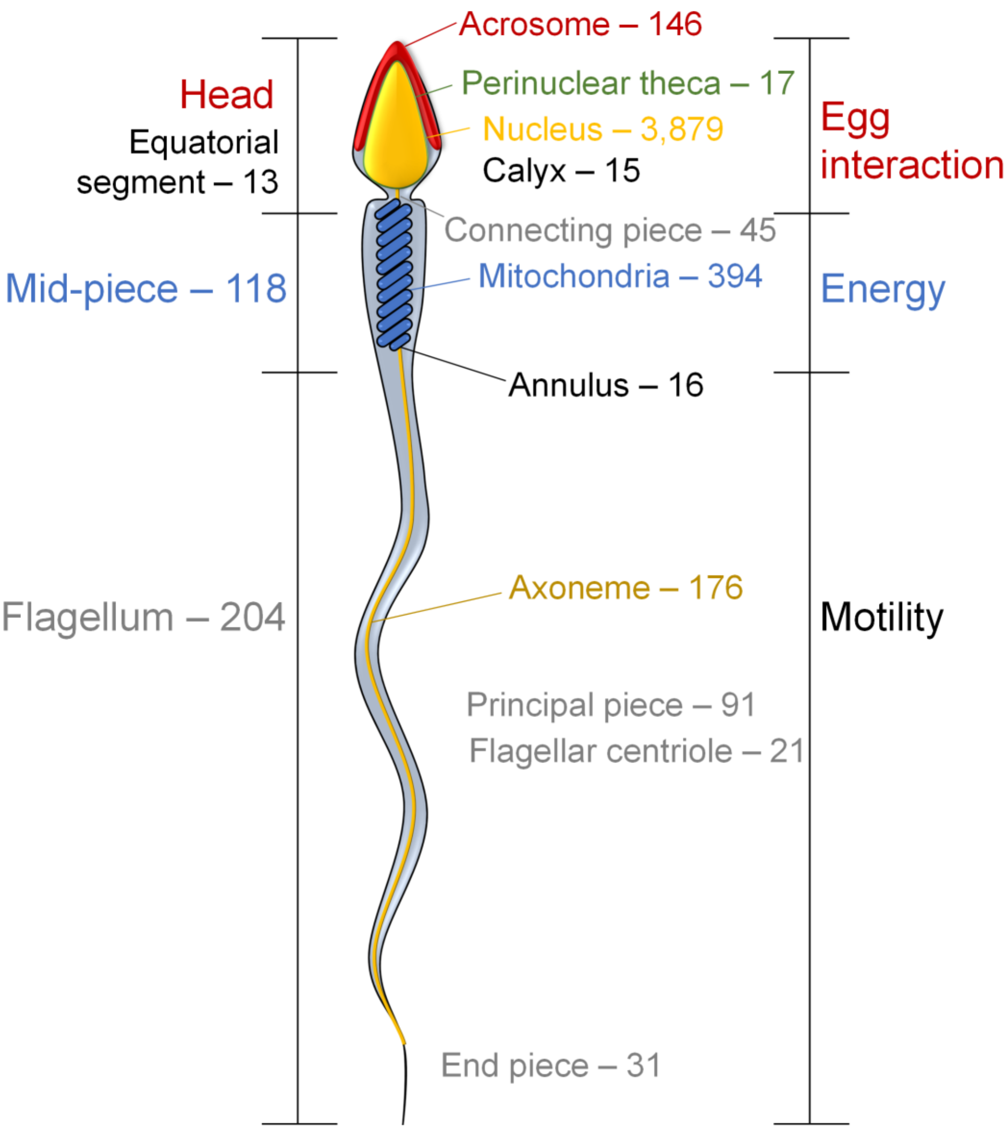

Figure S1: Sperm protein localization. Interrogation with UniProt maps proteins to their known sperm localizations.

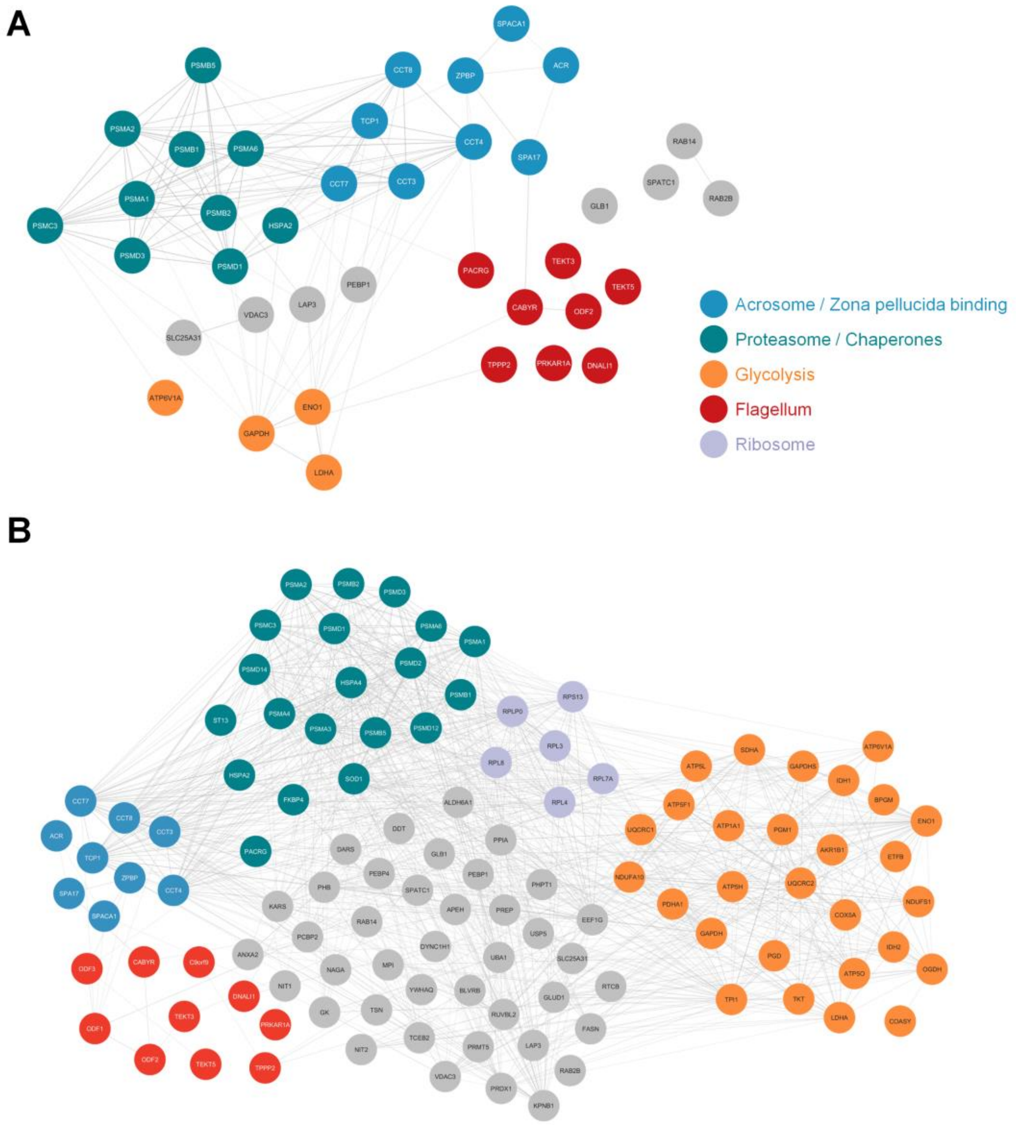

Figure S2: Protein-to-protein interaction networks of core sperm proteomes. STRING network analyses of the core sperm proteome at the (A) species (45 proteins) and (B) order (135 proteins) taxonomic levels, drawn and coloured with Cytoscape.

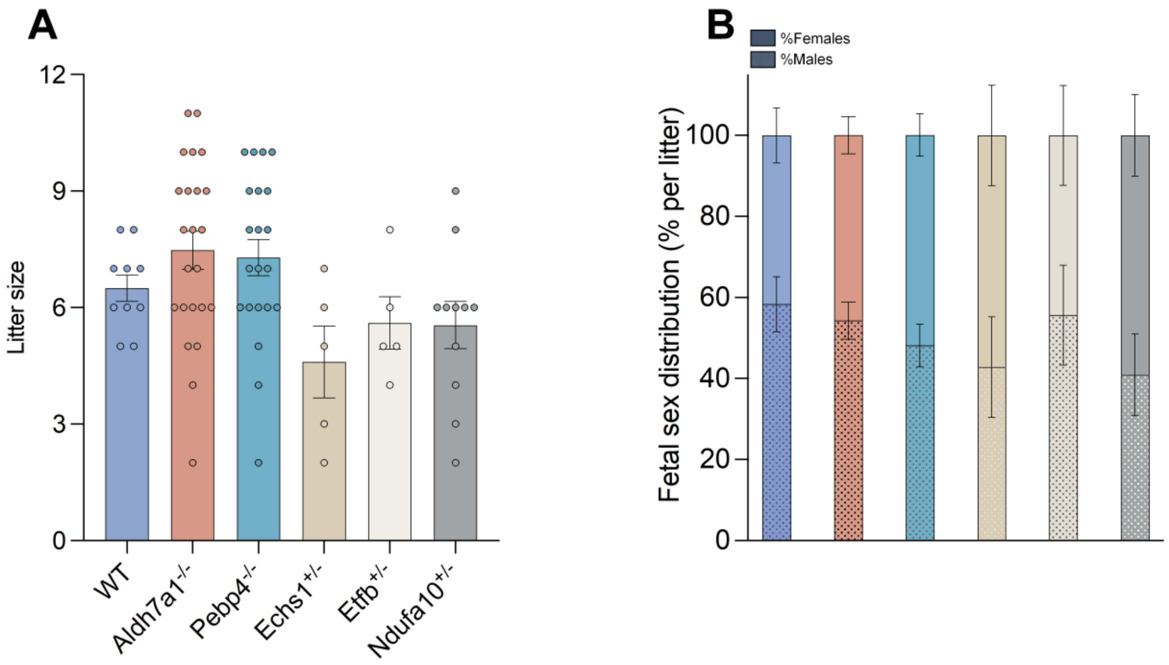

Figure S3: Knockout mouse model pregnancy outcomes. Following successful births, the (A) litter size and (B) foetal sex distribution was recorded.

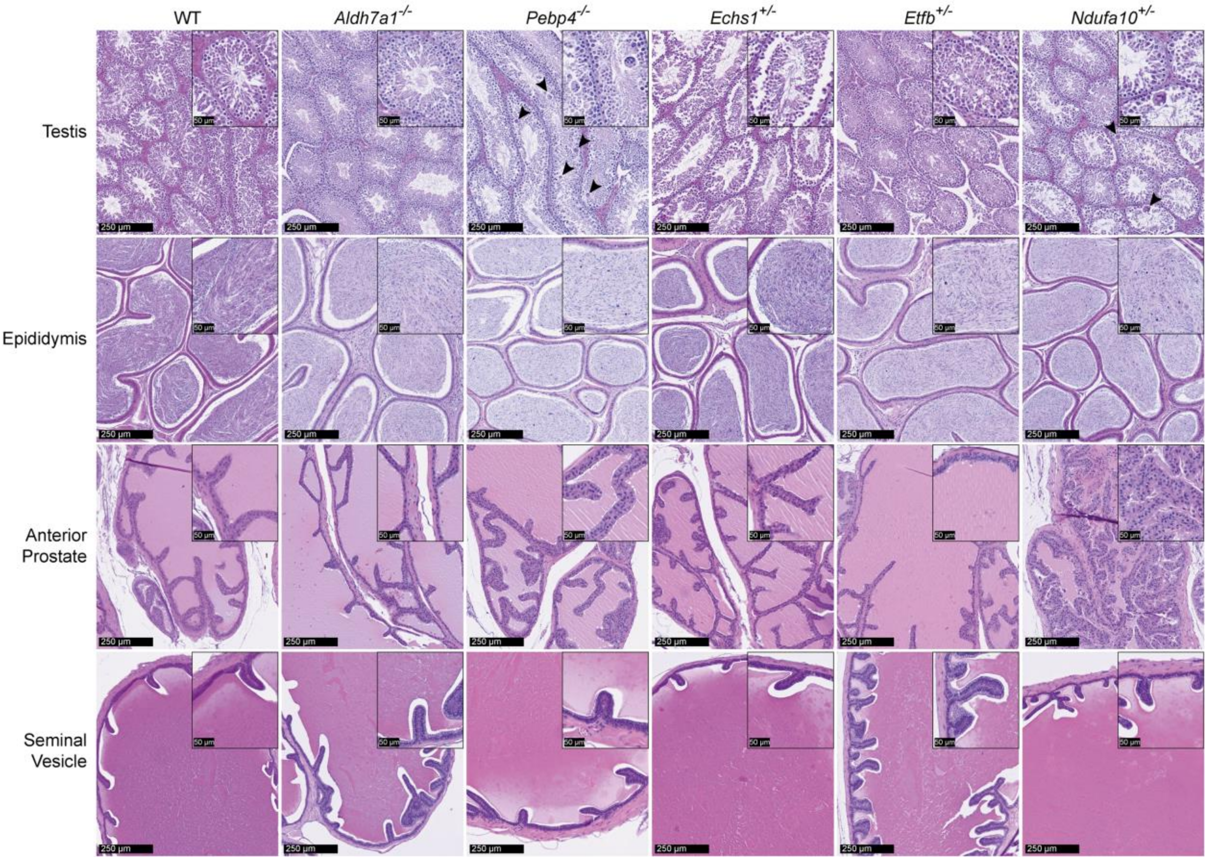

Figure S4: Histopathology of five KO models across the male reproductive tract. Representative images of H&E-stained sections from testis, cauda epididymis, anterior prostate and seminal vesicles of controls and knockout mice for the following genes: Aldehyde dehydrogenase 7 family member A1 (*Aldh7a1*), Phosphatidylethanolamine binding protein 4 (*Pebp4*), Enoyl-CoA hydratase, short chain 1 (*Echs1*), Electron transfer flavoprotein subunit beta (*Etfb*), and NADH:ubiquinone oxidoreductase subunit A10 (*Ndufa10*). Mice were 16 weeks old in all lines except for *Aldh7a1* (19 weeks old). Multinucleated giant cells (MGCs) are indicated by arrowheads. Scale bars are 250 µm and 50 µm.
